# Addressing barriers to physical activity among women: A feasibility study using social networking-enabled technology

**DOI:** 10.1177/2055207615583564

**Published:** 2015-05-05

**Authors:** Danielle Arigo, Leah M Schumacher, Emilie Pinkasavage, Meghan L Butryn

**Affiliations:** 1 Department of Psychology, The University of Scranton, 800 Linden Street, 205 Alumni Memorial Hall, Scranton, PA, USA; 2 Department of Psychology, Drexel University, 3141 Chestnut Street, Stratton 119, Philadelphia, PA 19104, USA

**Keywords:** Physical activity, women's health, technology, social support, social comparison

## Abstract

**Objective:**

Automated physical activity (PA) monitoring technology and associated social networks have potential to address barriers to PA, but have rarely been tested for PA promotion. This technology may be especially beneficial for women, who experience particular barriers to health-based social networking. The present study tested the feasibility and acceptability of pairing women as PA partners via technology-connected social networking. Social comparison (i.e. tendency to make self-evaluations relative to others) was examined as a mechanism of interest.

**Method:**

Overweight women (*n* = 12, *M*_age_ = 46, *M*_BMI_ = 32.60 kg/m^2^) used a PA sensor (daily wear = 93%) and communicated with an assigned partner (introduced via technology-connected social networking) for four weeks. Partners did not know one another prior to study enrollment.

**Results:**

PA meaningfully increased during the program, and was highest among participants who endorsed stronger (vs. weaker) tendencies toward social comparisons (*r* = 0.64). Participants identified several benefits of partner communication; however, some partners had difficulty initiating communication, and direct comparisons with partners were seen as unhelpful in this context. Most participants found the PA sensor beneficial, showed high compliance with daily wear recommendations, and reported an intent to continue using the PA sensor. Participants endorsed satisfaction with the program's approach and confidence in maintaining PA gains.

**Conclusions:**

These findings support the use of automated PA sensors and facilitated partner communication via social networking to promote PA among women. Insights from participant feedback identify specific avenues for program improvement; specifically, with respect to the potential difficulties of negative social comparisons.

## Introduction

Engaging in regular physical activity (PA) has multiple benefits, including reduced risk for cardiovascular disease,^1,2^ which remains a leading cause of death in the United States.^3^ Despite the known benefits of PA and myriad existing promotion efforts, most US adults do not engage in recommended levels (i.e. 150 minutes of moderate-intensity or 75 minutes of vigorous-intensity PA per week).^4^ Key barriers include low motivation,^5^ lack of time,^6^ and lack of social support for engaging in PA.^7,8^ Consequently, there is need for programs that address these barriers, using cost-effective, sustainable, and easily disseminable methods.

Advancements in internet-enabled PA monitoring technology, such as small sensors worn on the wrist, show promise for filling this gap. For example, sensors can reduce time and effort required for self-monitoring, and ensure that users receive accurate information about their progress, which is essential for effective behavior change.^9^ Progress is viewed in user-friendly, visually appealing formats (using web or mobile platforms), which may enhance and maintain motivation.^10^ To date, however, only research-grade versions of these devices have been tested, as adjuncts to in-person behavioral intervention; smaller and less expensive versions are commercially available, but their potential for supporting PA promotion is unknown. Previous tests of PA sensors have also occurred only in the context of weight loss trials.^11–13^ Assessing the utility of commercially available devices, specifically for PA, remains a key area of opportunity.

### Technology-supported social networking

Many available PA sensors also incorporate social networking platforms to connect users. Features include open-forum message boards and the opportunity to create private groups, in which users can see each other's PA progress on a “leaderboard.” Such platforms can facilitate beneficial processes such as social support, encouragement, and accountability to others.^14–16^ Online social networks may be particularly beneficial for receiving support, as users endorse greater perceived support than non-users.^17^ Despite these advantages, and although health-based social networking sites are free and easily accessible, many adults do not use them. Worry about privacy may create a significant barrier to using open forums; Americans who do not to use social networking sites endorse greater concerns about trust and privacy than those who do.^17^

In addition, sedentary, overweight women (more so than men) tend to cite shyness and embarrassment as factors that limit their physical activity.^18^ As engaging in social networking forums often involves self-initiation and personal disclosure, women who are physically inactive may experience added challenges to using these forums, especially those focused on PA. Women are also more likely to use social networking sites to maintain existing relationships than to form new relationships, and women are more likely to use direct messages than public forums.^19,20^ Such findings suggest that merely offering women the opportunity to participate in open forums – without aiding in the establishment of social connections – is unlikely to promote optimal engagement.

Team-based health promotion programs offer one alternative; these programs have shown real-world effectiveness^21^ and more robust outcomes than individual programs.^22,23^ To date, however, most of these programs require self-selection of teammates (i.e. signing up for a program as a team), necessitating that participants already know others who want to adopt healthy behaviors. Given that many women report lack of support as a barrier to PA, such programs do not meet the needs of this group. Providing women with a way to meet others interested in increasing PA, rather than requiring them to know someone who fits this criterion already, would offer a benefit beyond currently available programs. In addition, participants in organized programs that use social networking support tend to disengage over time,^24^ as group forums provide minimal individual accountability.

A novel alternative that could reduce engagement barriers for women is facilitating direct communication and accountability between users with similar goals, such as creating PA partnerships. The use of “peer coaches” to supplement face-to-face weight loss programs has shown promise,^25^ but has not been tested with PA promotion or online social networking. Creating PA partnerships via technology-supported social networking has additional benefits. It gives partners immediate and ongoing access to each other's objective PA data, which can improve the accuracy of partner feedback; it also increases the cost-effectiveness and disseminability of promotion efforts by reducing clinical contact.

### Social networking and social comparison

Online social networking also offers myriad opportunities to evaluate oneself relative to other users.^26,27^ Decades of research have shown that such *social comparisons* are a common and influential way of determining one's standing in important domains, including health.^21,28,29^ Comparisons are made toward selected (or available) *targets*, or others in the environment. Various experimental studies have demonstrated the importance of gender in both target selection and effect of a given comparison.^30,31^ Individuals are more likely to compare with and respond to targets of the same gender (in part due to the critical role of gender in identity),^32^ and this effect is particularly strong among women.^33^

Social comparisons may have either positive or negative consequences for PA. For example, comparisons to others perceived as “doing better” (i.e. upward comparisons) may increase motivation. The presence of similar, successful others demonstrates that improvement is possible (which may be especially helpful for women^34^), provides opportunities to learn from successful others,^35^ and may inspire friendly competition.^28,36^ However, these comparisons also may highlight the comparer's worse-off status, and thereby decrease motivation.^29,37^

To date, there has been little investigation of users' responses to comparison opportunities via PA-based social networking sites. Creating PA partnerships may encourage users to take advantage of social contact via PA-based social networking, and allows for exploration of social comparisons relevant to this emerging medium. Given that women show unique barriers to social networking engagement, and that gender plays a critical role in social comparisons, facilitating online PA partnerships between women may capitalize on the potential benefits of social networking-supported PA tracking.

### Aims of the present study

The present study was designed to pilot test an internet-based PA promotion program that combined evidence-based psychoeducation (delivered electronically) with automated PA self-monitoring and facilitated social connectivity (i.e. partner assignment and online communication). Whereas previous research with PA sensors has used research-grade PA sensors as a part of a weight loss program, the present study tested the use of smaller, commercially available PA sensors in the context of a program focused specifically on PA promotion. To our knowledge, this study is also the first to facilitate partnerships between individuals interested in PA who did not have existing relationships with one another.

As an initial test of these novel components, the program (a) recruited only women, and (b) was delivered over a limited time frame. As noted, we expected that social networking-enabled technology would have particular value for women. It is unclear which age range(s) would benefit most, however. For example, younger women may be interested in technology and social networking, but may not perceive these as novel; older women may have lower initial interest or greater difficulty learning to use the technology, but may derive equal or greater gains. In order to learn more about the type(s) of women who might engage in a technology-supported program, recruitment was open to women age 25–70. A four-week intervention period was chosen to maximize the quality and quantity of participant feedback, while allowing time for participants to benefit from various types of partner communication (such as encouragement, problem-solving, and supporting one another through challenges). Consequently, participants were not expected to show the type of linear PA progress or consistency that would be typical of longer and more intensive PA interventions.^38^

In addition, as scalability and disseminability were priorities for designing a new PA program, clinical contact was limited to baseline and end-of-treatment assessments. These in-person assessments did not include any discussion of steps or skills to use to increase PA, though participants were expected to select and achieve their own PA goals. Also, it was unclear to investigators how well participants would be able to identify the type(s) of partner communication or support they desired. To ensure that participants were introduced to basic skills to increase PA and could convey desired contact with partners *without* relying on face-to-face clinical contact, participants were provided with a brief overview of behavioral and communication skills in an online presentation format. We did not expect the skills module to confer considerable benefit beyond that of the PA sensor or partner.

Of primary interest were the *feasibility and acceptability* of (a) using automated, commercially available PA sensors with PA-related social networking capabilities among women, and (b) pairing female participants to provide support and accountability to one another (as both try to increase or maintain PA). To assess these outcomes, we examined objective and subjective use of a PA monitoring device, partner communication records, and participant feedback on each of these components. *Social comparison processes* were of secondary interest, and were examined with a validated self-report measure and participant feedback. The *pattern of PA change* was a tertiary and exploratory interest, as change over the brief intervention period was expected to be modest.

## Method

### Recruitment and participants

Study procedures were approved by the institutional review board at a mid-sized university in the northeastern United States. Women from the general community were recruited via print and electronic advertisements. Eligibility required age 25–70, the ability to engage in PA over the next month (i.e. no current injuries), and internet access to track PA and communicate with a partner. Eligibility was assessed in initial telephone interviews and in-person baseline visits. Participants were 12 adult women (six partner dyads). The average participant was Caucasian (75% of sample), 46 years old (*SD* = 13.09), and had a BMI of 32.60 kg/m^2^ (*SD* = 5.74) at baseline.

### Assessment procedures

#### Baseline

At in-person visits, staff assessed (a) height and weight using a Seca^©^ scale and stadiometer, (b) motivation and willingness to engage in PA, and (c) comfort with using the internet (for viewing an online psychoeducation module, monitoring PA, and communicating with a partner). To assist in pairing partners, potential participants were asked to describe their current PA levels. Those who were eligible and interested in enrolling were given a FitBit® Flex™ (FitBit Inc, 405 Howard Street, San Francisco, CA 94105) and instructions for accessing online psychoeducation materials. Participants also provided written informed consent and completed electronic self-report questionnaires.

### Mid-treatment

Participants completed a brief online questionnaire at the beginning of the third week of the intervention. Participants were asked to report on their experiences (e.g. goals, partner communication) midway through treatment to maximize the accuracy of retrospective recall.

### End-of-treatment

Participants completed an in-person assessment in the week after the intervention period ended. Each participant's height and weight were measured, the FitBit® was returned, and all FitBit® data were exported. Participants also completed online questionnaires and an exit interview. This interview was unstructured; participants were asked to describe what they liked and did not like about the program, and were prompted for as much detail as possible. Responses were categorized thematically by study staff and are presented as frequencies (i.e. the number of participants who discussed a particular topic).

### Intervention components

#### Online psychoeducation module

As noted, a web presentation was designed to prepare participants for increasing PA and communicating with their assigned partner. Participants were provided with login information for a secure website, where they could access the presentation in recorded webinar format: an interactive slide presentation with recorded narration. Narration was written and recorded by the first author, who is a licensed clinical psychologist with experience in delivering health behavior change interventions. The module differentiated lifestyle PA (i.e. steps per day) and structured/aerobic PA (i.e. minutes of moderate-to-vigorous PA [MVPA]), and presented three sets of skills: *cognitive-behavioral skills*;^39^ *acceptance-based skills*;^40^ and *communication skills*,^41^ described in Table 1. Cognitive-behavioral and communication skill sets reflected core components of established health behavior change programs.^39,42^ As empirical support for the utility of acceptance-based principles in health behavior change is growing,^43,44^ a small number of acceptance-based skills were included (e.g. engaging willingness).

**Table 1.** Online psychoeducation module domains and techniques.

Skill domain	Techniques	Examples
Behavioral	• Goal setting • Time management • Planning ahead • Lifestyle vs. structured (aerobic) PA • Target heart rate assessment	Effective Goal Setting • Specific, measurable, achievable, relevant, time-bound Time management: Rocks in a jar demonstration • Valued activities as “big rocks” that need to fit in the jar first (PA as “big rock”)
Acceptance	• Values clarification • Willingness to experience mild discomfort in order to meet PA goals • Mindful decision-making	Values: Why is PA important to you? • Connected to guiding principles (e.g. health) Willingness: PA “even if” motivation is low, because it is in the service of a value
Communication	• Identify desired modes of support (from significant others and PA partner) • Clearly explain how partner/significant others can be helpful	General communication • Describe desired behavior in specific, active terms • Clarify how the behavior will be helpful • Offer to compromise (if needed) Communicating with partner • Identify desired “help” from partner (e.g. remind of goals/holding accountable, offer suggestions for PA) • Share desired behaviors with partner • Discuss methods of communication (Community Board only vs. text, email, phone)

PA: physical activity.

Accompanying worksheets were available through the module website, to be printed and/or completed by participants on their private computers at home. These worksheets guided participants through identifying desired support from a partner and goal setting for each week of the intervention period. Goal setting guidance included an example of progress from 3000 to 10,000 steps per day, and 30 to 60 minutes of MVPA, over four weeks. Participants were encouraged to set goals relevant to the type(s) of activity they preferred (e.g. walking, cycling).

#### PA sensor

Participants received a FitBit® Flex™ and instructions for setup and care. FitBit® Community Boards were highlighted as the initial format for contacting assigned partners (described below). Participants were asked to wear the FitBit® without attempting to increase their PA on days prior to Week 1 of the program, which represented their “baseline” PA (*M* = 7 days for baseline). Participants were advised to wear the device every day during the intervention period, and to check their progress at least once before 5:00 pm each day (to ensure ample time to reach daily goals). The device's additional features (e.g. sleep tracking) were described as optional. Participants interested in cycling and swimming were encouraged to read the device's instructions specific to these activities. FitBit® PA reports were collected at end-of-treatment (EOT).

#### PA partner

Each participant who enrolled in the program was paired with another participant. Partners were assigned by research staff with three priorities in mind: age, reported starting level of PA (to maximize similarity between partners), and time of enrollment (to prevent long wait times for initial contact). As participants enrolled, age and starting level of PA were compared to existing (and as yet unpaired) participants; best matches were determined by staff judgment. At baseline, participants were encouraged to consider how partners might be helpful to each other (e.g. providing accountability, support, suggestions) and were directed to the online module's communication-relevant content (see Table 1).

After baseline visits, participants were alerted to their partner assignment via an email invitation to join a “private group” on the FitBit® website. This private group consisted of a secure web forum, which allowed partners to communicate and see each other's PA progress. The Community Board, which only partners could access, was a message forum that allowed thread posting and response. Participants were encouraged to use this forum to communicate with partners, though they were welcome to use alternative communication methods (such as email or texting) if both partners agreed to do so. Step totals for both partners were displayed on the Leaderboard, directly above the Community Board; each participant could click on Leaderboard options to see her partner's minutes of MVPA and total distance for the month.

### Materials and measures

#### Weekly goals

Participants reported on their PA goal(s) for each week of the program. Goals were set in number of steps per day, minutes of MVPA, and/or specific activities (e.g. going to the gym to use a particular machine). At EOT, participants reported whether they met some, all, or none of their goals for each week.

#### Partner communication

Partner communication was assessed in three ways. First, the *number* of Community Board posts per partner dyad were logged by research staff. Second, the *content* of partner messages was categorized thematically by research staff; both number and content of posts are described in Table 2. A third method relied on *self-report* of partner communication in order to capture any contact made outside of the Community Board. The self-report measure also assessed participant perceptions of communication, including both benefits of and barriers to partner interaction. Self-report was completed at mid-treatment and EOT, and referred to the past two weeks of communication (to limit the time frame of retrospective recall).

**Table 2.** Partner communication by dyad.

Dyad characteristics	Number of posts	Themes of communication
Ages 35 and 44 Both reported low PA at baseline	14 total (10 vs. 4)	• Congratulating each other on PA progress • Sharing tips for increasing PA
Ages 66 and 70 Both reported low PA at baseline	17 total (8 vs. 9)	• Checking in with each other to hear how each partner achieved her step totals • Sharing tips for increasing PA • Discussing other shared interests (e.g. gardening)
Ages 38 and 41 Both reported low to moderate PA at baseline	29 total (15 vs. 14)	• Problem-solving sensor difficulties • Sharing personal information (e.g. going on a vacation or a first date) • Congratulating each other on PA progress
Ages 47 and 52 Both reported low to moderate PA at baseline	16 total (9 vs. 7)	• Sharing tips for increasing PA • Checking in with each other and disclosing “forgetting” to wear the sensor
Ages 27 and 30 Both reported moderate PA at baseline	11 total (4 vs. 7)	• Reporting on daily activities and challenges • Describing use of psychological skills to motivate PA (e.g. willingness)
Ages 48 and 52 Both reported moderate PA at baseline	5 total (2 vs. 3)	• Describing goals and progress • Sharing PA-related plans and accomplishments (e.g. going to the gym rather than walking outside)

PA: physical activity.

#### FitBit® use

FitBit® wear (percentage of wear days) was determined objectively from exported data sets. A self-report measure, created for this study, also included items to assess the frequency with which participants viewed or used various FitBit® features (see Appendix A). Features of interest were PA totals and intensity, partner Leaderboards, and badges earned for milestones (e.g. 10,000 steps per day; see Appendix A). To inform future work in this area, we also assessed the use of FitBit's® non-PA capabilities such as journaling and tracking sleep, weight, or food intake. A final item assessed participants' purchase of (or intent to purchase) a FitBit® for personal use.

#### Treatment response and feedback

At mid-treatment, participants were asked to rate the effectiveness of the online psychoeducation module for (a) helping them to set goals for increasing PA, and (b) helping them to identify the type(s) of support they desired from their partners (see Appendix A). Items were rated from 1 (not at all) to 5 (very) with respect to effectiveness. Participants were also asked to provide written and verbal program feedback at EOT. Items regarding the program's effectiveness, participants' level of satisfaction with the program, and participants' confidence in their ability to continue their current PA were rated from 1 (not at all) to 5 (very). One item assessing whether participants would recommend the program to other women interested in increasing PA was rated from 1 (no) to 4 (yes, strongly). Participants were asked to provide specific suggestions for program improvements.

#### Social comparison

The Iowa-Netherlands Comparison Orientation Measure (INCOM) is a 23-item questionnaire that captures individual differences in the tendency to make social comparisons. The INCOM consists of three subscales: 11 items assess general comparison (e.g. “I often compare how my loved ones [boy or girlfriend, family members, etc.] are doing with how others are doing”); six items assess upward comparison (e.g. “When it comes to my personal life, I sometimes compare myself with others who have it *better* than I do”); and six items assess downward comparison (e.g. “When it comes to my personal life, I sometimes compare myself with others who have it *worse* than I do”). Items are rated on a scale of 1 (strongly disagree) to 5 (strongly agree). This measure has shown good psychometric properties;^45^ in the present study, Cronbach's alphas were 0.76 (general), 0.90 (upward), and 0.85 (downward).

#### Physical activity

PA was measured using a wireless PA sensor (the FitBit® Flex™), worn on the wrist each day. This device captures detailed information about several PA variables, including sedentary time, flights of stairs ascended, and the intensity of activity. These data are displayed on a web or mobile platform, and PA data are available for export from the web platform. FitBit® has been shown to capture reliable and valid information about PA.^46^ In the present study, only step counts and minutes of MVPA were used as outcomes.

### Data analysis

Descriptive statistics (e.g. means, frequencies) are presented to characterize participant response to intervention components, including daily sensor wear (from FitBit® reports), goal setting, use of sensor features, partner communication, and social comparisons between partners. Bivariate correlations were used to quantify associations between participants' self-reported comparisons at baseline and outcomes of interest (PA, use of FitBit® features). Finally, means and standard deviations were examined to describe change in PA during the intervention period.

## Results

### Online module and goal setting

All participants completed the online module (which took 45 to 60 minutes), and set specific, measurable goals using skills presented (e.g. “walking 5000 steps per day,” “taking the stairs at work,” and “biking 30 miles this week”). Average responses indicated that participants found the module useful for helping to set PA goals (*M* = 3.58 of 5), but less so for identifying the support desired from partners (*M* = 2.66 of 5). Of note, three participants set goals that increased PA every week; the remainder chose combinations of increases and maintenance over two or more weeks. All participants met all or some of their goals (e.g. met step goal but not MVPA goal) during the first two weeks, and the majority met all or some of their goals in the final two weeks (73% during week 3 and 82% during week 4). Retention over the intervention period was 100%.

### Feasibility and acceptability of PA sensors

FitBit® reports showed 93% compliance with program recommendations for daily wear (range 68–100%). During the first two weeks, all participants reported viewing their total PA via FitBit® profiles, and 11/12 endorsed viewing their PA intensity. Other commonly used features included badges (which mark milestones such as 10,000 steps in a day; 10/12) and tracking sleep (7/12). Half of participants (6/12) noted that they viewed their Leaderboard, which displayed their PA progress relative to their partner's. A smaller number of participants used food intake logging (3/12) and journal entry (2/12) capabilities.

Similarly, EOT responses showed that all participants viewed their total PA and PA intensity over the last two weeks of the program. Most participants continued to view their badges, track their sleep, and monitor their Leaderboard (7/12). A subset logged food intake and weight (3/12), using FitBit® or a compatible application. The majority reported intentions to purchase (or previous purchase of) a FitBit® for personal use (8/12).

### Feasibility and acceptability of partner assignment

Community Board posts showed that all participants posted at least once during each two-week period, and most posted more than twice in each period (11/12). On average, participants posted eight times (*SD* = 4.09), or twice per week. Frequency and type of communication, described by dyad, are displayed in Table 2. Primary themes of communication were identifying (or describing progress toward) goals for the week, disclosing difficulties, encouraging a partner to persist despite challenges, and inquiries about a partner's successes (e.g. requesting suggestions for increasing PA after observing a partner's progress).

#### Barriers to communication

Written and verbal feedback reflected both excitement about interacting with partners and difficulty establishing a schedule of communication. Several participants reported that they were hesitant to initiate conversation or “didn't know what to say” to a partner, and 11/12 suggested that the program build in additional structure for partner communication. For example, that participants could be prompted to share their responses to the online module handouts with their partners.

In addition, eight of 12 participants reported a desire for more frequent communication than they had during the program. Qualitative feedback showed that most participants were satisfied with partner communication at mid-treatment, and that partners were in consistent contact during the first two weeks of the intervention. Desire for greater communication did not emerge until EOT assessments, as partner contact became less consistent. Negative social comparisons, which also may have presented barriers to communication, are described below.

#### Benefits of communication

Despite difficulties with communication, participant feedback indicated that partnerships conveyed benefits, and the majority of participants wanted greater partner interaction. As expected, participants enjoyed receiving encouragement and being able to reach out to someone when they struggled to reach their goals (9/12), and a subset (4/12) explicitly acknowledged that they could have contacted their partner when they experienced challenges. Several participants (7/12) also referenced the benefits of accountability to their partners, receiving specific suggestions for increasing PA, and recognizing that they were “not alone” (i.e. that others face similar challenges to PA). All participants encouraged the continuation of partner-based programs, with adjustments to matching and communication processes.

### Participant response to treatment

Participants' average responses demonstrated that they found the program effective (*M* = 3.45 of 5) and were satisfied with the program's approach to increasing PA (*M* = 3.45 of 5). Overall, participants endorsed confidence in their ability to maintain their PA gains (*M* = 3.90 of 5). Participants also indicated that they would recommend the program to another woman interested in increasing PA (*M* = 3.36 of 4).

### Social comparison processes

Participants' general tendencies to make social comparisons were assessed at baseline (using the INCOM). Those who endorsed stronger overall tendencies toward comparison at baseline were somewhat more likely to report viewing their Leaderboards throughout the program (*t*[11] = 2.08, *p* = 0.06, *d* = 1.20). As discussed below, participants' daily steps and minutes of MVPA per week were determined from FitBit® records. Of note, individuals who endorsed stronger tendencies toward *upward* comparison reached higher peak MVPA minutes during the program (*r* = 0.64, *p* = 0.03).

In addition, the majority of participants (9/12) commented on comparisons with their partners (i.e. self-evaluations of PA progress relative to the assigned partner's progress) during follow-up interviews. Six speculated that their own (higher) PA levels may have been threatening to their partners, and three explicitly acknowledged feeling intimidated by their partners' objective PA levels. As noted, pre-treatment PA levels were self-reported at enrollment, and were considered during partner matching. At EOT visits, three participants stated that wearing a FitBit® showed the inaccuracy of their previous estimates (i.e. vast under- or overestimates), providing one possible explanation for discrepancies between partners' PA.

### PA change

FitBit® records showed that participants' starting activity levels averaged 5995 steps per day (*SD* = 3956) and 32.38 minutes of MVPA per week (*SD* = 27.64) at baseline. During the program, participants' highest PA weeks reached averages of 10,686 steps per day (*SD* = 3168) and 51 total minutes of MVPA (*SD* = 29.74) (see Table 3 for PA for each participant). Participants' PA did not change in a linear fashion, as several participants peaked before Week 4 of the program. Peak weeks did show meaningfully higher step totals and MVPA minutes than baseline, however. For example, baseline steps per day were far below the daily recommended level of 10,000 steps,^4^ whereas this level was achieved during peak weeks.

**Table 3.** Physical activity during the four-week intervention period.

		Average steps per day						Total MVPA minutes per week				
Participant		Baseline	Week 1	Week 2	Week 3	Week 4		Baseline	Week 1	Week 2	Week 3	Week 4
1		9007	12119	10351	10908	11122		60	21	18	27	15
2		5218	3989	5073	6312	2973		19	10	18	25	7
3		4826	4740	3685	3429	8644		17	13	6	7	40
4		8775	10263	8326	11506	9978		7	5	4	6	5
5		10718	13483	12104	13844	9206		49	74	58	73	44
6		7876	6602	9054	9135	10296		49	29	33	40	60
7		13793	11628	11102	13741	17611		83	74	57	77	109
8		6000	6277	4223	5165	3057		15	19	11	14	6
9		9917	8843	1095	6785	5065		38	37	36	26	13
10		2974	12297	12578	9465	6027		4	41	91	25	13
11		9000	9578	5894	7158	8519		20	52	17	46	45
12		5280	9310	7159	7722	7623		10	40	24	27	16

Note: Physical activity totals based on FitBit® records for each week.

MVPA: moderate-to-vigorous physical activity.

## Discussion

The use of automated PA tracking technology and associated online social networking capabilities has the potential to address barriers to PA, including those that seem particularly relevant to women.^5,47^ Yet engaging women in their use – particularly the use of social networking – remains challenging. The present study examined the feasibility of increasing engagement by (a) offering access to commercially available, automated PA sensors, and (b) facilitating partnerships between women interested in increasing or maintaining PA. Previous team- and partner-based health promotion programs have required participants to enroll as a group,^21^ or have created partnerships between members of a face-to-face weight loss group,^25^ with some success. In contrast, women in the present study did not previously know each other, and were paired based on mutual interest in PA.

Findings show that participants were able to increase their PA over four weeks, with minimal clinical contact. Changes were not always progressive, however. First, some participants were fairly active at baseline, and set goals to maintain their levels of PA. Second, it is likely that such a brief intervention period did not allow participants to create consistent PA habits,^38^ and there is an opportunity to examine this process over a longer follow-up. Participants endorsed overwhelmingly positive responses to the PA sensor and showed high compliance with daily wear recommendations. The majority planned to continue using the system after the end of the program, demonstrating the benefit of introducing women to automated sensors.

Participants also had positive responses to social networking-based partner communication; receiving support, encouragement, and ideas for increasing PA were cited as benefits. Most participants indicated that they wanted more contact with their partners, lending support to the desirability and potential benefit of partner-based programs. Interestingly, desire for more frequent communication emerged during the last two weeks of the program, and some participants recognized that they could have instigated more communication. These responses indicate more than a simple mismatch between women who wanted frequent versus infrequent contact. Rather, they underscore previously observed challenges to initiating social connections in this population.^18,19^ In the present sample, “not knowing what to say” to a partner – someone known to have a similar interest in PA – was cited as a barrier to ongoing contact.

### Technology-based social networking and social comparison

The present study also examined two aspects of social comparisons which are known to affect motivation for effortful activities.^48,49^ The first was participants' self-reported tendencies to make comparisons, as measured at baseline. Even in a small sample, we observed that participants who began the study with a stronger (vs. weaker) tendency to make upward comparisons also reached higher peak MVPA minutes. Comparisons considered in baseline responses occurred prior to partner communication, however, and did not capture comparisons with assigned PA partners. The second was frequency of viewing the FitBit® Leaderboard, which showed each partner's accumulated PA, and was visible each time a participant logged in (FitBit® was selected for the present study, in part, to explore the use of this feature, and any associated comparisons). Although upward comparisons may be useful under certain circumstances,^29^ several participants endorsed negative responses to upward comparisons with partners (i.e. partners were perceived as much “better” at increasing or maintaining PA).

Negative responses to comparisons, such as those observed here, can be triggered by “unavoidable” comparisons with a single other who was perceived as highly dissimilar in a valued domain.^35,50^ Such responses may have created additional barriers to partner communication. These findings are consistent with previous work, which has shown that participants in health behavior change programs responded more positively to peer coaches than to expert coaches.^25,51^ Partners in the present study were paired to maximize similarity in starting PA level; inaccurate estimates of PA limited partner congruence, highlighting the utility of automated tracking technology in this population.

Although partner assignment has rarely been used in interventions, previous laboratory experiments have shown that motivation for PA increases when assigned partners are judged based on the performance of the lowest-scoring partner.^22^ Neither participant tendency toward competitiveness nor induced competition was employed in the present study, but may be useful avenues for future work. In addition to increasing motivation, introducing an element of competition between dyads (rather than between partners) may improve partnership cohesiveness and individuals' perceptions of similarity to their partners,^52,53^ thereby reducing the likelihood of negative responses to partner comparisons.

## Conclusions

Together, findings from this pilot study provide four key insights. First, participants were able to set and achieve PA goals with minimal – and remote – intervention. Second, positive responses to the PA sensor and high compliance with daily wear recommendations demonstrate the feasibility of using such technology to promote PA among women. Third, participants were able and eager to communicate with women who share their health goals via technology-enabled social networking sites – particularly when communication was facilitated via partner assignment. Finally, specific improvements to the current program design, such as those described below, may maximize the potential of technology-enabled social networking sites and facilitated partnerships to promote PA among women.

The current study relied on participant self-reports of baseline PA to assign partners; FitBit® reports (which included baseline PA) were not collected until the end of treatment. Using objectively assessed PA from a “baseline” period to match partners could ensure that partners themselves are similar, and reduce the likelihood of partner comparisons that decrease motivation.^54^ Including this step could also limit any potential reactivity to initiating PA assessment.^55^ Future work could examine the utility of considering characteristics such as communication style for optimizing partner matching, and creating a formal system or algorithm for matching participants. Participants also had difficulty initiating conversation with their partners. Providing additional structure to guide partner communication (e.g. prompts on Community Boards) could remove perceived barriers to connection between partners, and a single, face-to-face meeting at the beginning of the program could be used to establish rapport.

In addition, although participants were able to successfully set and achieve PA goals with minimal guidance, providing structured PA prescriptions to those who begin the program at lower levels of PA may facilitate progressive increases. It also remains unclear to what degree the online skills module was beneficial, relative to the PA sensor and partner aspects of the current program. Follow-up work should include more rigorous differentiation of the efficacy of each program component. Finally, increasing the length of the intervention may reveal additional insights, as participants would have more time to receive the full benefits of progressive goal setting, automated PA monitoring, and partner communication. The present findings indicate that larger-scale tests of such improvements are warranted, and show promise for promoting PA among women.

## Appendix A: FitBit® and treatment response measures

### FitBit® use

Please indicate whether or not you have used these features of FitBit**®** in the PAST TWO WEEKS:

**Table:**

	NO	YES
Viewing my amount of physical activity (e.g. steps or distance)		
Viewing the intensity of my physical activity		
Viewing my partnership physical activity leaderboard		
Viewing my badges (or other milestones)		
Logging food intake in FitBit®		
Logging food intake in an app that connects to FitBit®		
Logging weight		
Tracking sleep		
Writing a journal entry		

### At end-of-treatment only

**Have you purchased, or do you intend to purchase, a FitBit® for your own use?**

1. I have already purchased a FitBit®
2. I intend to purchase a FitBit®
3. I intend to purchase a different activity tracker
4. I do not intend to purchase an activity tracker

### Treatment acceptability questionnaire

#### Mid-treatment

How effective was the online module for helping you *set physical activity goals*?

1. Not at all effective
2. A little effective
3. Somewhat effective
4. Effective
5. Very effective

How effective was the online module for helping you identify the kind of support you wanted from your partner?

1. Not at all effective
2. A little effective
3. Somewhat effective
4. Effective
5. Very effective

### End-of-treatment

How effective was the program for helping you increase your physical activity?

1. Not at all effective
2. A little effective
3. Somewhat effective
4. Effective
5. Very effective

How satisfied were you with the approach we used to help you increase your physical activity?

1. Not at all satisfied
2. A little satisfied
3. Somewhat satisfied
4. Satisfied
5. Very satisfied

Please indicate how confident you are that, over the next three months, you will be able to keep up the level of physical activity you are doing now (check one item):

1. Not at all confident
2. A little confident
3. Somewhat confident
4. Confident
5. Very confident

Would you recommend this treatment to another woman wishing to increase her physical activity?

1. No, I would not recommend it
2. I'm not sure
3. Yes, I'd recommend it, with some hesitation
4. Yes, I'd recommend it strongly
